# Evaluating composite PRF–fat–matrix strategies for soft-tissue augmentation: A preliminary screening study in a porcine model

**DOI:** 10.1016/j.jpra.2026.01.052

**Published:** 2026-02-06

**Authors:** Martin Fiala, Pavel Rotschein, Ladislav Plánka, Edita Jeklová, Viktoriia Zaporozhets, Alica Hokynková, Petr Šín

**Affiliations:** a Department of Paediatric Plastic Surgery, Department of Paediatric Surgery, Orthopaedics and Traumatology, University Hospital Brno, Brno, Czech Republic; b Faculty of Medicine, Masaryk University, Brno, Czech Republic; c Veterinary Research Institute, Brno, Czech Republic

**Keywords:** Autologous fat grafting, Platelet-rich fibrin (PRF), Acellular dermal matrix (ADM), Composite lipografting, Porcine model, Wound healing

## Abstract

Soft-tissue augmentation using lipografting remains challenging in constrained recipient environments characterized by scarring, limited vascularity, restricted volume, and high demands on shape stability and tissue quality. Secondary correction of the soft palate in velopharyngeal insufficiency (VPI) represents a clinically relevant example of such conditions.

A porcine dorsal skin model was designed to simulate a vascularly compromised recipient environment using standardized full-thickness defects covered in selected groups with split-thickness skin grafts (STSG). In this model (3 × 3 cm defects), spontaneous healing, STSG alone, autologous fat grafting, fat combined with platelet-rich fibrin (PRF), and fat + PRF combined with an acellular dermal matrix (ADM; MatriDerm®), a non-dermal acellular matrix (ANDM; DuraGen Plus®), or an absorbable gelatin matrix (AGM; Cutanplast®) were compared.

Macroscopic healing was evaluated at 3 and 5 weeks, focusing on exudation, contraction, and geometry preservation. Fat + PRF showed reduced exudation and improved surface quality compared with other composite approaches. Matrix-based combinations were technically feasible but associated with increased exudation, while AGM demonstrated better preservation of defect geometry.

These findings indicate that the proposed porcine model is suitable for detecting differences between composite lipografting strategies and support further experimental optimization prior to clinical translation.

## Introduction

Secondary correction of the soft palate for velopharyngeal insufficiency (VPI) after cleft palate repair remains a challenging clinical problem, particularly when revision is required in scarred and hypoplastic tissue. Standard surgical approaches include pharyngeal flap, sphincter pharyngoplasty, and Furlow palatoplasty.^1^ In selected cases, palatal re-repair may be feasible, whereas nonsurgical options include prosthetic devices or speech therapy.^2^

For mild to moderate VPI, augmentation techniques such as autologous fat grafting (lipofilling) have been reported since the early 2000s.^1^ Reported advantages include minimal invasiveness, repeatability, and a favorable safety profile, with potential improvements in resonance and phonation. However, unpredictable graft resorption remains a major limitation, often necessitating repeated injections. These approaches build on the presence of adipose-derived stem and progenitor cells within lipoaspirate, which contribute to the regenerative potential of fat grafts.^5^

**Figure 1:**
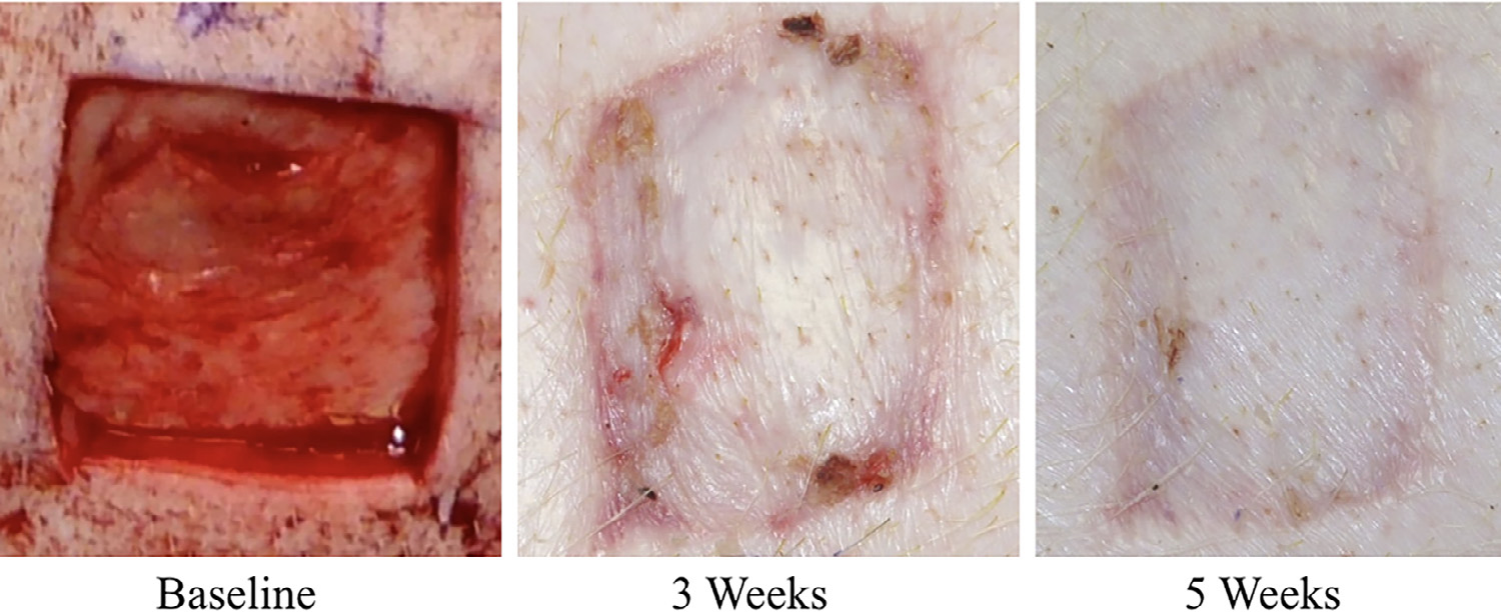
Representative macroscopic appearance of a standardized full-thickness defect treated with autologous fat combined with platelet-rich fibrin and covered with split-thickness skin graft, shown at baseline, during healing at 3 weeks, and at the final healing endpoint at 5 weeks.

**Figure 2:**
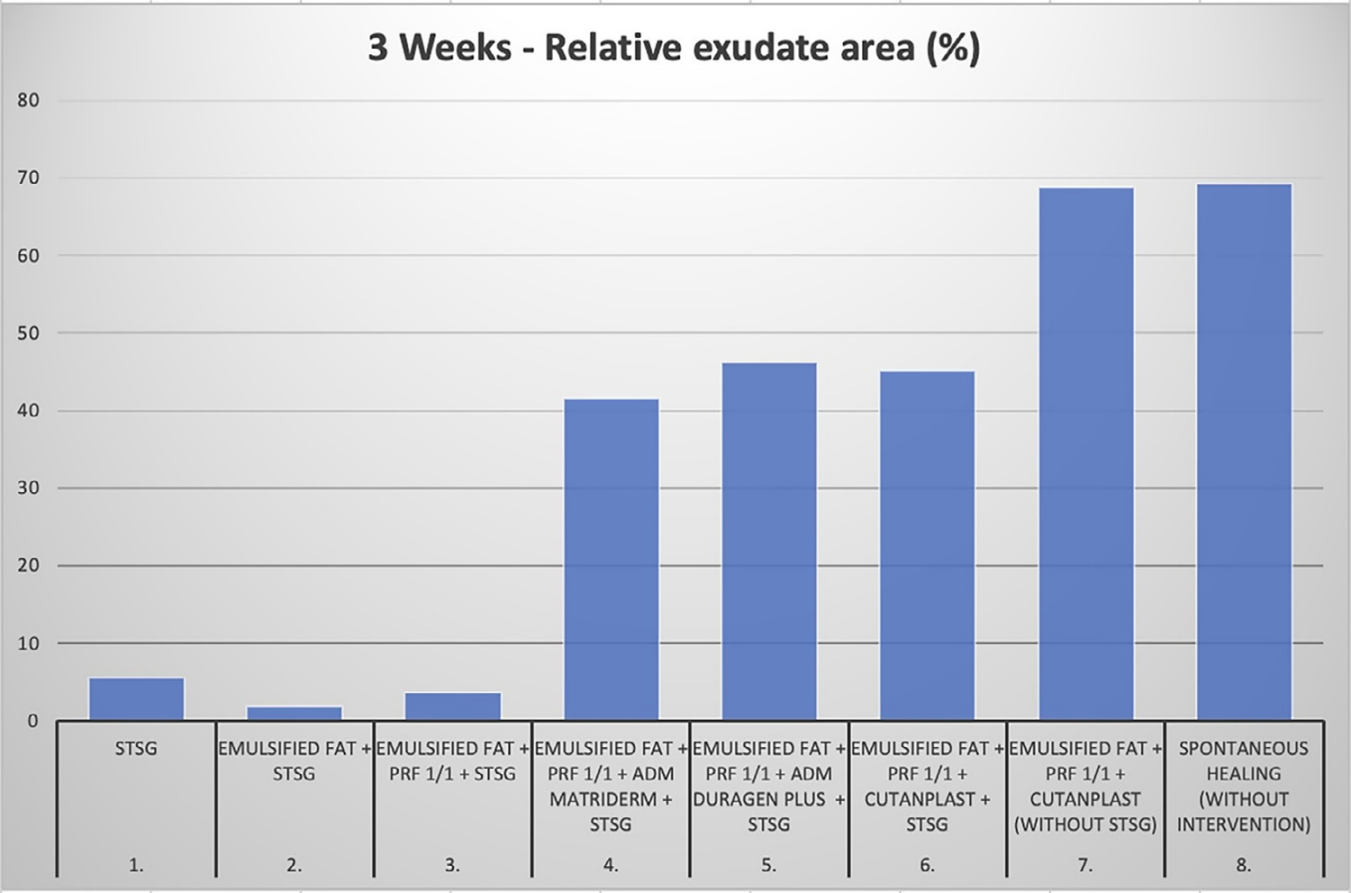
Quantitative assessment of relative exudation area (% of defect area) at 3 weeks. Additional macroscopic documentation and quantitative analyses are provided in the Supplementary Material.

To improve volume retention and tissue quality, experimental strategies have focused on combining autologous fat with platelet-rich fibrin (PRF)^2^^,^^3^ or with scaffold-based adjuncts, including acellular dermal matrices (ADM), non-dermal acellular matrices (ANDM), and absorbable gelatin matrices (AGM). Consequently, any augmentation strategy must provide stable volume, preserve shape fidelity, and remain well tolerated in this anatomically and functionally constrained environment (Figure 1, Figure 2).

## Materials and methods

The dorsal porcine model was selected to allow preliminary testing of multiple specimens under standardized and reproducible conditions.^4^ The composite defect-and-fill design, including the use of split-thickness skin grafts (STSG), was intended to simulate the clinical environment of secondary lipografting in scarred soft-palate tissue, characterized by reduced vascularity and limited perfusion.

Standardized 3 × 3 cm full-thickness defects were created on the porcine dorsum under general anesthesia. Experimental groups included spontaneous healing, STSG alone, autologous fat grafting, fat combined with platelet-rich fibrin (PRF), and fat + PRF combined with an acellular dermal matrix (ADM; MatriDerm®), a non-dermal acellular matrix (ANDM; DuraGen Plus®), or an absorbable gelatin matrix (AGM; Cutanplast®).

ADM preserves native dermal collagen architecture, whereas ANDM provides a fibrillar type I collagen scaffold with intermediate mechanical properties. AGM represents a gelatin-based matrix with reduced structural organization.

Fat was harvested by mini-liposuction and mechanically emulsified by syringe-to-syringe transfer without filtration or enzymatic digestion. Injectable platelet-rich fibrin (i-PRF) was prepared from autologous venous blood by low-speed centrifugation and used immediately.

Macroscopic wound healing was assessed using standardized photographic documentation. Exudation was quantified as the relative exudation area using ImageJ software. Data analysis was descriptive, reflecting the exploratory nature of the study. Detailed quantitative data and additional macroscopic documentation are provided in Supplementary Tables S1–S3 and Supplementary Figures S1–S4.

## Results

The combination of autologous fat and platelet-rich fibrin (PRF) demonstrated the most consistent macroscopic healing pattern, with surface characteristics approaching those of intact skin. Fat grafting alone showed a similar but less pronounced effect. Both fat alone and fat + PRF were associated with reduced exudation compared with matrix-based combinations.

Within the acellular matrix groups, material-dependent differences were observed. ANDM (DuraGen Plus®) showed the highest degree of exudation, whereas ADM (MatriDerm®) and AGM (Cutanplast®) induced more moderate tissue reactions. ADM provided improved surface texture, while AGM showed better preservation of defect geometry. Matrix-based augmentation without STSG coverage resulted in poor integration and prolonged exudation.

Overall, composite lipografting was technically feasible across all tested variants, with distinct differences in macroscopic healing response between treatment groups.

## Discussion

This preliminary porcine study demonstrates that the proposed defect model is feasible and suitable for the experimental evaluation of composite lipografting strategies. The model was able to detect meaningful differences in macroscopic healing response between treatment groups.

Among the evaluated approaches, the combination of autologous fat and platelet-rich fibrin (PRF) showed the most favorable surface appearance and lowest degree of exudation, indicating a distinct signal compared with other composite variants. In contrast, composite lipografting incorporating acellular matrices was associated with a stronger tissue response, reflected by increased exudation.

STSG coverage proved to be a critical factor for successful integration, as matrix-based augmentation without coverage resulted in prolonged exudation and poor incorporation. These findings emphasize the influence of material selection and coverage strategy on the healing response in vascularly compromised recipient environments.

This study is limited by its exploratory design, small sample size, and use of a dorsal porcine skin model, which does not fully replicate the anatomy or functional loading of the soft palate. Nevertheless, the model provides sufficient translational relevance to support further refinement and optimization of composite lipografting strategies under controlled experimental conditions.

## Conclusion

This pilot porcine study demonstrates that the proposed model is suitable for the experimental evaluation of composite lipografting strategies. The model was able to identify meaningful differences in macroscopic healing response between treatment groups, with autologous fat combined with platelet-rich fibrin showing the most favorable profile. These findings support further optimization of composite lipografting approaches and justify continued experimental investigation prior to clinical application.

## Funding

Supported by the 10.13039/501100003243Ministry of Health of the Czech Republic – conceptual development of research organization (FNBr, 65269705).

## Ethical approval

Approved by the institutional animal ethics committee.

## Declaration of competing interest

The authors declare that they have no known competing financial interests or personal relationships that could have appeared to influence the work reported in this paper.

## Appendix Supplementary materials

Image, application 1

Supplementary Figure S1 Representative macroscopic appearance of standardized fullthickness defects at baseline and during healing at 4 weeks across different treatment groups. Image, application 2

Supplementary Figure S2 Representative macroscopic appearance of standardized fullthickness defects at the final endpoint during biopsy harvest. Image, image 3 dummy alt text

Supplementary Figure S3 Quantitative assessment of roundness at 3 weeks across experimental groups. Image, image 4 dummy alt text

Supplementary Figure S4 Quantitative assessment of roundness at 5 weeks across experimental groups. Image, image 5 dummy alt text
