# Supplementary material for: Evaluating composite PRF–fat–matrix strategies for soft-tissue augmentation: A preliminary screening study in a porcine model
Source: JPRAS Open. 2026 Feb 6;49:149–53. doi: 10.1016/j.jpra.2026.01.052 (PMC12969459; doi:10.1016/j.jpra.2026.01.052)
Supplement: Supplementary file 1 [file mmc1.docx]

Supplementary Table S1. Quantitative wound parameters at 3 weeks (relative measurements): surface descriptors. Values are presented as mean ± SD unless otherwise indicated. For groups with n = 1, single values are shown.

| Group | Relative defect area (a.u) | Relative exudate area (a.u) | Exudate  (%) | Defect perimeter (a.u) | Exudate perimeter (a.u) | Defect roundness |
| --- | --- | --- | --- | --- | --- | --- |
| STSG | 708444.50 ± 302435.93 | 40211.00 ± 22640.14 | 5.49 ± 0.85 | 3442.84 ± 965.20 | 1640.62 ± 569.40 | 0.65 ± 0.20 |
| Emulsified fat + STSG | 799806.00 ± 278813.62 | 14532.00 ± 1476.44 | 1.90 ± 0.48 | 3640.35 ± 697.99 | 518.67 ± 34.62 | 0.61 ± 0.16 |
| Emulsified fat + PRF 1/1 + STSG | 956269.50 ± 508792.32 | 34689.00 ± 19414.32 | 3.60 ± 0.12 | 3870.60 ± 938.01 | 1363.87 ± 866.44 | 0.62 ± 0.12 |
| Emulsified fat + PRF 1/1 + ADM Matriderm + STSG | 966663.00 ± 7891.31 | 401082.00 ± 191300.67 | 41.57 ± 20.13 | 5367.91 ± 1288.81 | 4065.02 ± 1152.89 | 0.60 ± 0.05 |
| Emulsified fat + PRF 1/1 + ADM DuraGen Plus + STSG | 1323813.00 ± 349776.03 | 597588.00 ± 49235.85 | 46.26 ± 8.51 | 5023.37 ± 617.46 | 4894.34 ± 816.98 | 0.71 ± 0.12 |
| Emulsified fat + PRF 1/1 + Cutanplast + STSG | 1273467.00 ± 330488.98 | 559170.00 ± 30580.95 | 45.12 ± 9.31 | 5549.60 ± 1338.10 | 6085.58 ± 92.64 | 0.84 ± 0.10 |
| Emulsified fat + PRF 1/1 + Cutanplast (without STSG) | 693024.00 | 477057.00 | 68.84 | 4916.02 | 2777.70 | 0.78 |
| Spontaneous healing (without intervention) | 711702.00 | 492792.00 | 69.24 | 5113.15 | 3754.19 | 0.66 |

Supplementary Table S2. Quantitative wound parameters at 3 weeks (relative measurements): shape descriptors. Values are presented as mean ± SD unless otherwise indicated. For groups with n = 1, single values are shown.

| Group | Exudate roundness | Defect solidity | Exudate solidity | Defect circularity | Exudate circularity |
| --- | --- | --- | --- | --- | --- |
| STSG | 0.52 ± 0.18 | 0.93 ± 0.06 | 0.09 ± 0.02 | 0.75 ± 0.10 | 0.18 ± 0.02 |
| Emulsified fat + STSG | 0.58 ± 0.1 | 0.96 ± 0.00 | 0.96 ± 0.06 | 0.75 ± 0.02 | 0.68 ± 0.02 |
| Emulsified fat + PRF 1/1 + STSG | 0.42 ± 0.14 | 0.94 ± 0.03 | 0.48 ± 0.58 | 0.77 ± 0.05 | 0.31 ± 0.22 |
| Emulsified fat + PRF 1/1 + ADM Matriderm + STSG | 0.79 ± 0.09 | 0.7 ± 0.24 | 0.63 ± 0.36 | 0.46 ± 0.22 | 0.39 ± 0.35 |
| Emulsified fat + PRF 1/1 + ADM DuraGen Plus + STSG | 0.66 ± 0.04 | 0.89 ± 0.03 | 0.61 ± 0.09 | 0.65 ± 0.01 | 0.32 ± 0.08 |
| Emulsified fat + PRF 1/1 + Cutanplast + STSG | 0.68 ± 0.33 | 0.81 ± 0.04 | 0.57 ± 0.25 | 0.53 ± 0.12 | 0.19 ± 0.02 |
| Emulsified fat + PRF 1/1 + Cutanplast (without STSG) | 0.90 | 0.64 | 0.94 | 0.36 | 0.78 |
| Spontaneous healing (without intervention) | 0.70 | 0.72 | 0.82 | 0.34 | 0.44 |

Supplementary Table S3. Healing parameters at 5 weeks (mean ± SD).

| Group | % wound closure (vs. baseline) | n | Mean area (cm²) | Circularity (mean) | Roundness (mean) | Solidity (mean) |
| --- | --- | --- | --- | --- | --- | --- |
| STSG | 17.28 | 2 | 7.45 | 0.69 | 0.57 | 0.89 |
| Emulsified fat + STSG | -1.05 | 2 | 9.09 | 0.74 | 0.54 | 0.93 |
| Emulsified fat + PRF 1/1 + STSG | -4.76 | 2 | 9.43 | 0.69 | 0.54 | 0.89 |
| Emulsified fat + PRF 1/1 + ADM Matriderm + STSG | 57.87 | 2 | 3.79 | 0.32 | 0.45 | 0.57 |
| Emulsified fat + PRF 1/1 + ADM DuraGen Plus + STSG | 24.97 | 2 | 6.75 | 0.55 | 0.50 | 0.81 |
| Emulsified fat + PRF 1/1 + Cutanplast  + STSG | 25.54 | 2 | 6.70 | 0.52 | 0.76 | 0.75 |
| Emulsified fat + PRF 1/1 + Cutanplast (without STSG) | 60.67 | 1 | 3.54 | 0.26 | 0.45 | 0.47 |
| Spontaneous healing (without intervention) | 73.98 | 1 | 2.34 | 0.14 | 0.34 | 0.33 |
