# Supplementary figures and images for: Evaluating composite PRF–fat–matrix strategies for soft-tissue augmentation: A preliminary screening study in a porcine model
Source: JPRAS Open. 2026 Feb 6;49:149–53. doi: 10.1016/j.jpra.2026.01.052 (PMC12969459; doi:10.1016/j.jpra.2026.01.052)

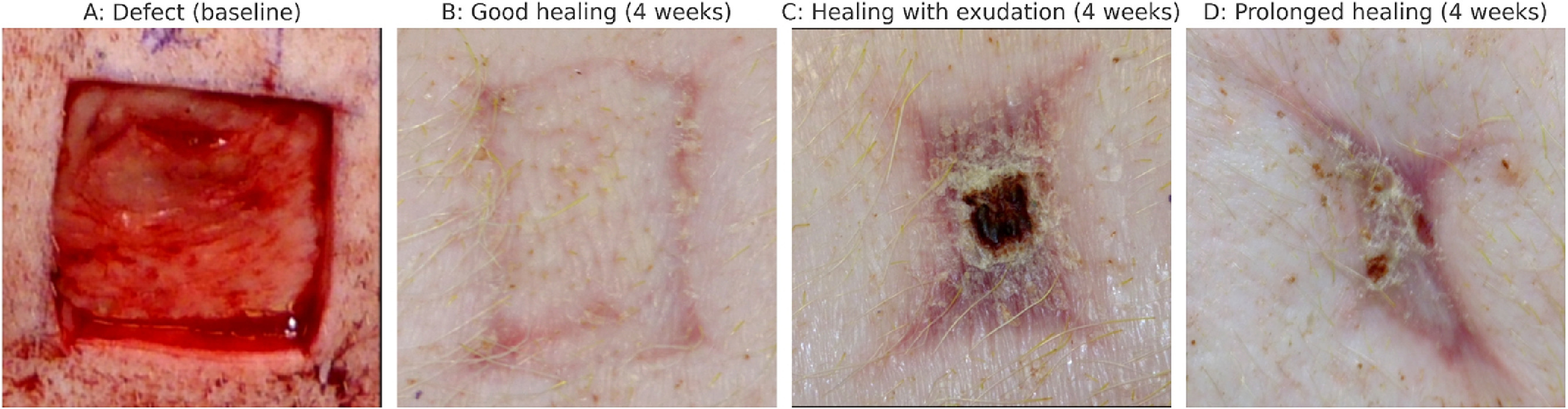

Supplement: Supplementary Figure S1 — Representative macroscopic appearance of standardized fullthickness defects at baseline and during healing at 4 weeks across different treatment groups. [file mmc2.jpg]

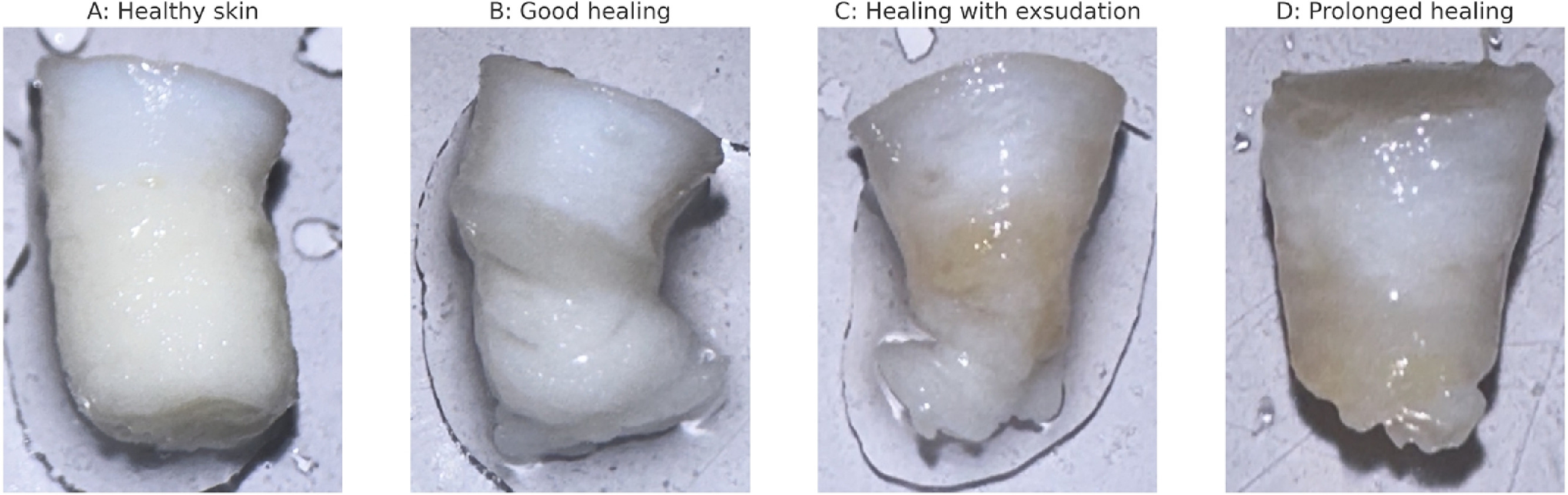

Supplement: Supplementary Figure S2 — Representative macroscopic appearance of standardized fullthickness defects at the final endpoint during biopsy harvest. [file mmc3.jpg]

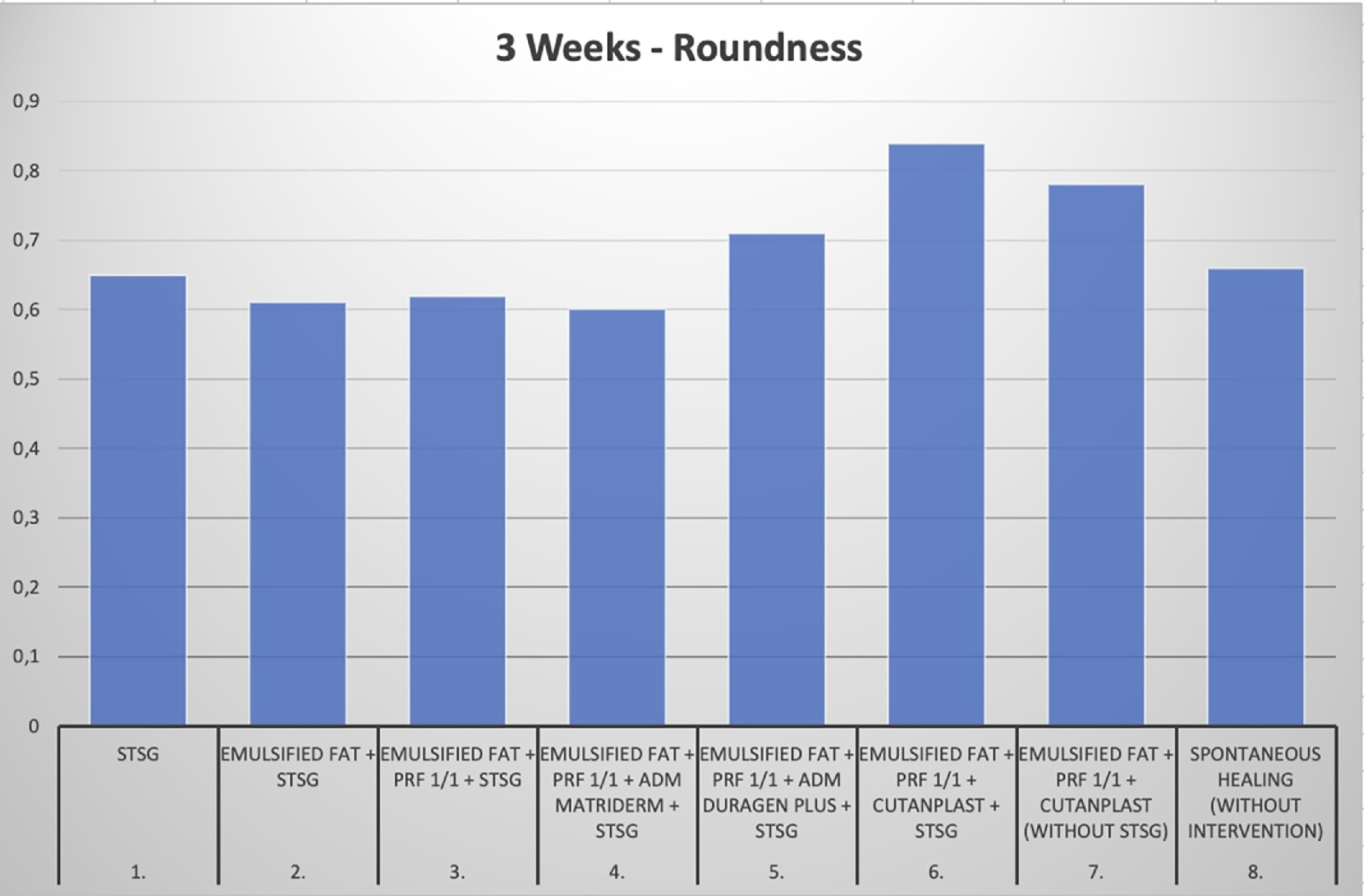

Supplement: Supplementary Figure S3 — Quantitative assessment of roundness at 3 weeks across experimental groups. [file mmc4.jpg]

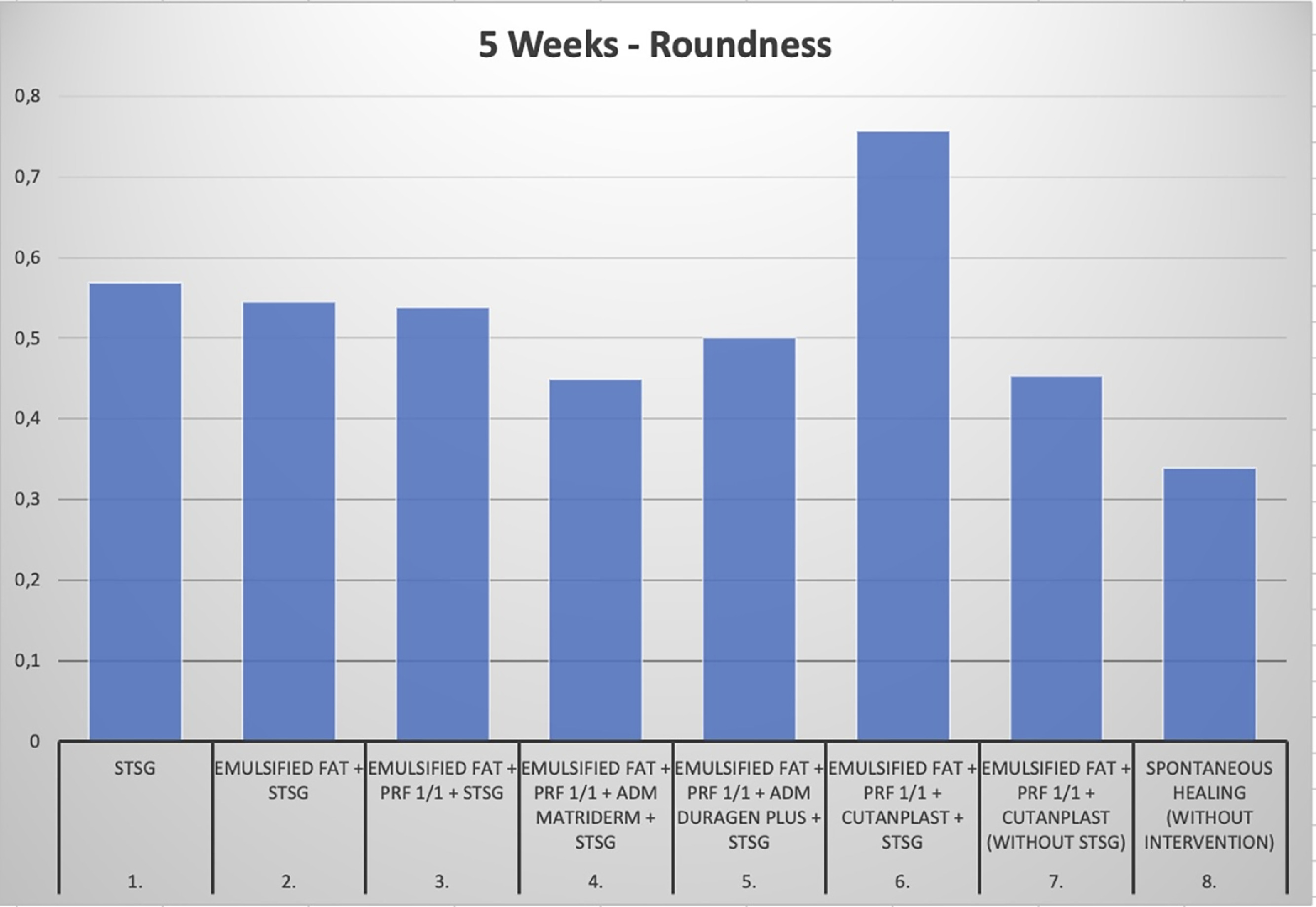

Supplement: Supplementary Figure S4 — Quantitative assessment of roundness at 5 weeks across experimental groups. [file mmc5.jpg]
